# Methods for multiancestry genome‐wide association study meta‐analysis

**DOI:** 10.1111/ahg.12572

**Published:** 2024-07-18

**Authors:** Chuan Fu Yap, Andrew P. Morris

**Affiliations:** ^1^ Centre for Genetics and Genomics Versus Arthritis, Centre for Musculoskeletal Research, Division of Musculoskeletal and Dermatological Sciences The University of Manchester Manchester UK

**Keywords:** genetic variation, genome‐wide association studies, meta‐analysis, software, multiancestry

## Abstract

Genome‐wide association studies (GWAS) have significantly enhanced our understanding of the genetic basis of complex diseases. Despite the technological advancements, gaps in our understanding remain, partly due to small effect sizes and inadequate coverage of genetic variation. Multiancestry GWAS meta‐analysis (MAGMA) addresses these challenges by integrating genetic data from diverse populations, thereby increasing power to detect loci and improving fine‐mapping resolution to identify causal variants across different ancestry groups. This review provides an overview of the protocols, statistical methods, and software of MAGMA, as well as highlighting some challenges associated with this approach.

## 1 INTRODUCTION

The advent of genome‐wide association studies (GWAS) has revolutionized our understanding of the genetic underpinnings of complex diseases (Sollis et al., 2023). Initially hindered by high costs and limited sample sizes, technological advancements have now enabled the examination of millions of single nucleotide polymorphisms (SNPs), uncovering modest‐effect genetic signals across a plethora of human diseases and traits. Despite these advances, a significant challenge persists in the form of the “missing heritability” gap, attributable to small effect sizes of SNPs on complex traits and poorer coverage of genetic variation with lower allele frequency (Visscher et al., 2012).

To increase sample size, and the power to detect new loci of more modest effect and lower allele frequency, contemporary GWAS are conducted in large collaborative consortia where association summary statistics are aggregated through meta‐analysis, without the need for sharing of individual‐level genotype and phenotype data. Examples of such international collaborative efforts include the Psychiatric Genomics Consortium, the Genetic Investigation of Anthropometric Traits Consortium, the Global Lipids Genetics Consortium, and the Type 2 Diabetes Global Genomics Initiative (Kanoni et al., 2022; Suzuki et al., 2024; Yengo et al., 2022; Zhang et al., 2023). Meta‐analysis circumvents the challenge of data sharing restrictions, such as those imposed by General Data Protection Regulation (GDPR), which prevents the aggregation of individual‐level data in “joint analysis”. It is important to emphasize that for common variants, joint analysis and meta‐analysis strategies have similar power (Lin & Zeng, 2010), therefore providing a robust approach to assemble large sample sizes without the restrictions of individual‐level data sharing.

Early GWAS meta‐analyses were predominantly undertaken in individuals of European ancestry. However, the field has shifted toward the integration of multiancestry GWAS, increasing sample sizes and providing a broader representation of genetic diversity, thereby enhancing the power and precision to detect and localize disease‐causing genetic variants across diverse populations. An important distinction in this context is between population of origin/ethnicity and genetic ancestry; population of origin/ethnicity are social constructs grouping individuals based on shared biological characteristics and culture, while genetic ancestry is a statistical construct derived from DNA information (Banda et al., 2015; Lewis et al., 2022). Expanding genetic diversity in GWAS is essential because European ancestry individuals do not encompass all genetic variation, thereby limiting detection of causal variants that are of higher frequency in other ancestry groups, which may implicate genes and biological pathways that are important drivers of disease across more diverse populations. Moreover, poor transferability of polygenic scores derived from European ancestry GWAS to other ancestry groups severely limits opportunities for clinical translation (Fatumo et al., 2023; Kamiza et al., 2022; Xiang et al., 2024), underscoring the importance of multiancestry analyses for more equitable disease prediction across diverse populations.

In this review, we provide an overview of the protocols and methods for multiancestry GWAS meta‐analysis, which we refer to as MAGMA. We begin by considering data harmonization and quality control (QC). We then review the statistical methods employed for MAGMA, considering heterogeneity between studies, and highlighting the software tools available for MAGMA. Finally, we look ahead to future directions and remaining challenges in the field.

## 2 DATA HARMONIZATION AND QUALITY CONTROL

The integration of multiancestry GWAS necessitates rigorous data harmonization and QC procedures to ensure the validity and reliability of meta‐analysis findings. Harmonizing phenotype measurements across studies and standardizing results is pivotal in MAGMA. This involves ensuring consistency in the distribution of effect sizes and effect allele frequencies across GWAS within the same ancestry group. This process facilitates the pooling of association summary statistics across GWAS, thereby augmenting the sample size and enhancing statistical power. The harmonization process requires adherence to detailed protocols to maintain consistency in study design features and analytical methods across GWAS (Winkler et al., 2014). The nuances of this process include ensuring the uniformity of covariate definitions and adjustments, such as age and gender, across all datasets (where appropriate). In the context of MAGMA, adjustments for covariates that may differ by ancestry groups should also be considered, such as socioeconomic status, environmental risk factors, and population‐specific health behaviors. It is essential to account for these factors in MAGMA to ensure accurate and equitable genetic insights across diverse populations. However, in the interpretation of findings, it is important to consider that adjustment for covariates that have a genetic component can potentially lead to false‐positive associations due to collider bias (Aschard et al., 2015).

Genotype imputation plays a crucial role in MAGMA by enhancing the resolution of SNP coverage across ancestry groups. The choice of reference panels impacts the quality of imputation and the density of SNPs available for MAGMA. The optimal reference panel may vary by ancestry group, and could include ancestry‐specific reference panels (Zhang et al., 2011), or make use of the Trans‐Omics Precision Medicine (TOPMed) study, which brings together the largest and most genetically diverse collection of whole‐genome sequence data to date for imputation (Taliun et al., 2021).

Traditionally, GWAS have been undertaken in samples of individuals from the same ancestry group. However, with the advent of population biobanks, GWAS often encompass increased genetic diversity that spans multiple ancestry groups. In this context, individuals are typically clustered into distinct ancestry groups based on their genetic makeup and self‐reported population of origin/ethnicity. Each ancestry group is then analyzed separately before MAGMA because admixed individuals may inflate false‐positive error rates if population structure is not adequately accounted for in the analysis and can impact the quality of imputation depending on the distribution of ancestry groups in the GWAS (Huang & Tseng, 2014).

## 3 THE IMPACT OF HETEROGENEITY IN MAGMA

Heterogeneity in the effects of SNPs across GWAS can arise, particularly in the context of MAGMA, because of variability in the patterns of linkage disequilibrium (LD) between ancestry groups (Liu et al., 2013). When testing association with a SNP that is in LD with a causal variant, we would expect to see differences in effect sizes between ancestry groups that are driven by variability in the structure of LD due to population history. Specifically, we would expect to see a larger effect size in an ancestry group for which the LD is stronger between the SNP and causal variant than in an ancestry group where the LD is weaker. These differences in LD structure between ancestry groups are beneficial for fine‐mapping causal variants because they break down the correlation in association signals due to LD that are observed within a single‐ancestry group (Zaitlen et al., 2010). Other factors that can lead to heterogeneity include interactions with SNPs that vary in allele frequency between ancestry groups, interactions with environmental risk factors that vary in exposure between ancestry groups, and technical artifacts (such as imputation quality) that are impacted by genetic ancestry. Unless these factors are directly modeled or otherwise accounted for in GWAS analyses, they can result in heterogeneity in effect sizes between ancestry groups. For example, if a causal variant interacts with an environmental exposure, and this interaction is not modeled in the GWAS analysis, the marginal effect of the SNP will be observed only in those ancestry groups for which the environmental exposure is present.

A commonly used measure to assess heterogeneity in SNP effects between GWAS is Cochran's *Q* statistic. However, with few GWAS, the test has low power to detect heterogeneity, and with many GWAS, it may overstate the importance of minimal differences in SNP effects (Kuchenbaecker et al., 2019). An alternative metric is the *I* ^2^ statistic, which is less sensitive to the number of GWAS than Cochran's *Q* statistic, and which provides an estimate of the proportion of observed variance in SNP effects due to heterogeneity rather than chance. Heterogeneity in MAGMA can be informative, revealing important biological insights, such as differential genetic effects across ancestry groups or biological settings. For example, association signals for type 2 diabetes have been shown to be enriched for heterogeneity in SNP effects between ancestry groups, which can be mostly explained by differences in the distribution of body mass index between East Asian ancestry individuals and those from other ancestry groups (Suzuki et al., 2024). Forest plots are suggested as a heuristic method for visualizing and understanding heterogeneity in SNP effects across GWAS (Begum et al., 2012).

The traditional approach to GWAS meta‐analysis is under a fixed‐effects model, which assumes no heterogeneity in SNP effects across GWAS, and may therefore not be optimal in the context of MAGMA. In contrast, random‐effects meta‐analysis allows for heterogeneity in SNP effects between GWAS, while metaregression models heterogeneity due to a covariate, such as genetic ancestry. In the next section, we contrast these standard methods for meta‐analysis to specialist software developed for MAGMA (Table 1).

**TABLE 1:** Commonly used meta‐analysis software for aggregating genome‐wide association studies (GWAS) results across ancestry groups.

Software name	Description	Method(s)	Features/advantages	Citations
GWAMA	Open‐source software for GWAS meta‐analysis	Fixed‐effects and random‐effects meta‐analysis	• Handles large datasets, • Error trapping, • Graphical summaries, • Corrects for population structure, genomic control, •Heterogeneity testing	Mägi and Morris (2010)
MAMA	Combines GWAS summary statistics from multiple populations	Cross‐ancestry GWAS meta‐analysis with generalized method of moments	• Efficient information sharing, • Bias reduction, • Type‐1 error control, • Linkage disequilibrium (LD) patterns correction, • Computational efficiency	Patrick et al. (2021)
MetaSoft	Introduces a new random‐effects model for GWAS meta‐analysis	Novel random‐effects meta‐analysis (RE2)	• Higher statistical power, • Accurate false‐positive rate control, • Efficient handling of heterogeneity, • Confounding correction	Han and Eskin (2011)
MANTRA	Software tool for multiancestry meta‐analysis of GWAS	Multiancestry meta‐analysis with Bayesian partition model	• Tailored for multiancestry GWAS, • Bayesian approach, • Improved detection and localization, • Robust to genetic heterogeneity	Morris (2011)
MR‐MEGA	Tool for multiancestry metaregression of GWAS	Multiancestry metaregression with linear regression framework	• Specialized for multiancestry GWAS, • Genetic variation (due to ancestry) incorporation, • Enhanced power and resolution	Mägi et al. (2017)
METAL	Designed for efficient meta‐analysis of GWAS	Sample size‐based meta‐analysis, inverse–variance‐based meta‐analysis	• Computational efficiency, • flexible input formats, error detection and correction, • genomic control correction	Willer et al. (2010)
PLINK	Widely used tool for GWAS and genetic epidemiology	Fixed‐effects and random‐effects meta‐analysis	• Covers wide range of tools for GWAS, including quality control, association testing and more	Purcell et al. (2007)

## 4 STATISTICAL METHODS AND SOFTWARE FOR MAGMA

### 4.1 Basic principles

The primary aim of meta‐analysis is to aggregate association summary statistics across GWAS to estimate a combined SNP effect size and/or *p* value for association. This integration not only bolsters the power to detect genetic effects but also helps in exploring heterogeneity between GWAS—a critical aspect of MAGMA, as described in the previous section. By aggregating summary statistics across GWAS from diverse ancestry groups, meta‐analysis enables the examination of a broader spectrum of genetic variants than would be possible within an individual ancestry group because of increased genetic diversity. The input for meta‐analysis for each SNP is a set of association summary statistics for each GWAS, which typically include the estimated effect size (aligned to an effect allele), the standard error of the effect size estimate, and *p* value for association. This section will briefly describe the general statistical models used for meta‐analysis, with further details provided in the Appendix.

### 4.2 Fixed‐effects and random‐effects meta‐analysis

The two most common methods for meta‐analysis implement inverse–variance weighting of effect sizes across GWAS under a fixed‐effects model or a random‐effects model. These methods provide a mean SNP effect estimate across GWAS, weighted according to the inverse of the variance of the effect size, but which differ according to how the weights are calculated. From the resulting statistics, a *p* value for association can be calculated (details provided in the Appendix). The inverse of the variance gives greater weight to effects that are more precisely estimated, which can depend on many factors, including allele frequency, sample size, and imputation quality.

In a fixed‐effects meta‐analysis, it is assumed that a single true effect size is shared across all GWAS. This model is particularly effective when the GWAS being combined are homogenous in nature, that is, they are similar in their design, trait/disease definition, genetic ancestry, and environmental exposures. Under a fixed‐effects model, the weight attributed to an effect size estimate is simply the inverse of the within‐study variance of that effect size estimate.

The random‐effects model, in contrast, recognizes the existence of variability in the true effect sizes across GWAS, i.e. that they follow a distribution with between‐study variance. This model is therefore particularly pertinent in MAGMA where heterogeneity in SNP effects across GWAS from different ancestry groups is more likely. The random‐effects model accounts for both within‐study variance and between‐study variance, thereby increasing power to detect association in the presence of heterogeneity in SNP effects between GWAS. The standard random‐effects model adapts the weights used in fixed‐effects meta‐analysis as the sum of the estimated within‐ and between‐study variances (DerSimonian & Laird, 1986). In the context of MAGMA, the random‐effects model has been further adapted to the RE2 approach, which accounts for the fact that the between‐study variance is zero under the null hypothesis of no association (Han & Eskin, 2011).

### 4.3 Metaregression

In the context of MAGMA, random‐effects meta‐analysis has the advantage of allowing for heterogeneity in SNP effects across GWAS but does not assume any “structure” to the heterogeneity. We would expect, for example, that SNP effects from GWAS undertaken in the same ancestry group will be less heterogeneous than those from GWAS undertaken in different ancestry groups. Metaregression extends the simple fixed‐ and random‐effects weighting schemes by examining the influence of study‐level factors on heterogeneity. This method uses a linear regression framework to model SNP effect estimates, weighted by the inverse–variance of the estimate, as a function of study‐level covariates. For MAGMA, this could simply be an indicator variable of ancestry group. However, the MR‐MEGA metaregression instead includes principal components derived from SNP allele frequency differences across GWAS to model the genetic diversity as continuous and multidimensional (Mägi et al., 2017). The MR‐MEGA metaregression implements a fixed‐effects model after accounting for principal components, i.e. there is no heterogeneity in SNP effects after allowing for genetic ancestry. However, the metaregression model can easily be adapted to allow for random effects (Dias et al., 2012; Stanley & Doucouliagos, 2015). In simulations of GWAS from five major ancestry groups, it was found that MR‐MEGA with three axes of genetic variation as covariates, demonstrated greater power to detect associations compared to both fixed‐ and random‐effects model when heterogeneity was present between ancestry groups under a range of scenarios (Mägi et al., 2017). The gains in power were greatest when effect of the causal variant was limited to one ancestry group.

### 4.4 Alternative methods for MAGMA

Fixed‐ and random‐effects meta‐analysis approaches (including their implementation in metaregression) are standard statistical methods to aggregate effect size estimates across studies and are not specific to MAGMA. However, methods and software have been developed that specifically address the heterogeneity in SNP effects anticipated in MAGMA.

MANTRA models heterogeneity in SNP effects between GWAS under a Bayesian partition model of the genetic similarity between studies Morris (2011). This method considers the expected similarity in SNP effects among GWAS from the same ancestry group while allowing for greater heterogeneity between different ancestry groups. This is achieved by clustering GWAS based on their genetic distance and observed SNP effects. MANTRA assumes the number of clusters of GWAS is unknown. With one cluster, the model is equivalent to fixed‐effects meta‐analysis. In contrast, when each GWAS belongs to its own cluster, the model is equivalent to random‐effects meta‐analysis. MANTRA provides a Bayes’ factor in favor of association for a SNP, together with the posterior probability of heterogeneity in SNP effects between GWAS. It was demonstrated, using simulated data, that MANTRA had greater power to detect associations and improve fine‐mapping resolution over fixed‐ and random‐effects models. This advantage was greatest for complex heterogeneity patterns that were correlated with ancestry.

MAMA employs generalized method of moments (GMM) that accommodate differences between ancestry groups in conditional effects, allele frequencies, and LD to address the expected heterogeneity in MAGMA Patrick et al. (2021). GMM is a parameter estimation method that relies on moment conditions. For MAMA, there is a moment condition that GWAS within the same ancestry group share the same “true” effect size. Under GMM, MAMA estimates effect sizes within and between ancestry groups. An important feature of MAMA is that the moment conditions depend on LD matrices that are computed from ancestry‐specific reference panels, allowing for optimal adjustment of SNP effects across ancestry groups prior to meta‐analysis. When compared to MR‐MEGA and MANTRA in simulations, while MAMA had lower statistical power, it demonstrated lower bias across all LD correlation levels and maintained more controlled type‐1 error rates. This robustness makes MAMA effective in scenarios in which heterogeneity in SNP effects is common across ancestry groups.

### 4.5 Meta‐analysis software for GWAS

Meta‐analysis can be conducted in standard statistical software. However, in the context of GWAS meta‐analysis, there are typically millions of SNPs to process, and there can be challenges due to strand differences between studies (which can cause a mismatch of alleles or errors due to palindromic AT/GC SNPs). Table 1 provides an overview of commonly used software tools for MAGMA, highlighting their key features and advantages.

## 5 FUTURE DIRECTIONS AND REMAINING CHALLENGES

The burgeoning field of MAGMA has expanded our understanding of the genetic contribution to complex human traits and diseases, leveraging the power of genetic diversity to enhance fine‐mapping resolution, identify ancestry‐specific associations and reveal novel loci (Kichaev & Pasaniuc, 2015; Lam et al., 2019; Meng et al., 2024; Walters et al., 2019). For example, in a recent MAGMA of type 1 diabetes (T1D) across three ancestry groups, the *NRP1* locus was associated with the disease in African and American ancestry groups but not in the European ancestry group, suggesting that this genomic region is more relevant to disease risk for individuals of non‐European ancestry. The study also found human leukocyte antigen (HLA) alleles that decreased risk for the American ancestry group but increased risk for the European ancestry group, potentially reflecting differences in HLA haplotype structure between ancestry groups (Michalek et al., 2024). The latest MAGMA in major depression identified 53 novel loci associated with the condition and achieved improved fine‐mapping resolution by reducing the number of candidate causal variants (Meng et al., 2024). In a recent MAGMA of type 2 diabetes across six ancestry groups, 145 novel loci associated with the disease were identified, and significant enrichment of ancestry‐correlated heterogeneity in SNP effects was detected through metaregression (Suzuki et al., 2024). In rheumatoid arthritis, MAGMA across five ancestry groups identified 34 novel loci, and multiancestry fine‐mapping identified a putative causal variant at the *LEF1* locus, which is of interest as *LEF1* has a role in CD4^+^ T cells, which are relevant to rheumatoid arthritis biology (Ishigaki et al., 2022).

The inclusion of GWAS from diverse ancestry groups improves the transferability of polygenic scores (Cavazos & Witte, 2021; Choudhury et al., 2022; Martin et al., 2019). For example, a polygenic score for coronary heart disease developed through MAGMA achieved better performance than a polygenic score derived using a single‐ancestry approach (Smith et al., 2024). In type 2 diabetes, polygenic scores developed using SNPs identified from MAGMA explained a larger proportion of the liability‐scale variance than those derived from ancestry‐specific GWAS, particularly into those ancestry groups with the smallest sample sizes (Mahajan et al., 2022). Analyses across African and European ancestry groups that included several diseases demonstrated that polygenic scores developed with multiancestry data achieve better performance in admixed individuals than single‐ancestry scores (Cavazos & Witte, 2021). Although the performance of polygenic scores benefits from MAGMA, there are still challenges in generalizing results for some ancestry groups due to the complexities of LD patterns and differences in environmental exposures (Kamiza et al., 2022; Kuchenbaecker et al., 2019; Topriceanu et al., 2024). Further developmental work is required to optimize methods for multiancestry polygenic scores.

Large sample sizes are paramount in MAGMA, as they help to navigate through the intracacies of differing LD structures and allele frequency distributions across genetically diverse GWAS. The challenge lies in aggregating sufficient data across varied ancestry groups to attain clear and generalizable genetic insights. Addressing the lack of diversity in genetic studies is a multifaceted challenge, which involves not only augmenting the participation of diverse ancestry groups in research but also ensuring equitable access to data resources. Continued reliance on limited demographic groups hampers our holistic understanding of disease biology and may inadvertently widen global health inequities. The concentration of GWAS data predominantly in European ancestry individuals poses a significant limitation, highlighting the critical need for an increased focus on under‐represented and underserved ancestry groups, including across continental Africa, South and Central America, the Middle East, and Oceania (Mills & Rahal, 2020; Popejoy & Fullerton, 2016).

There is a pressing need for the development and implementation of sophisticated statistical methods for GWAS analysis that are tailored to multiancestry contexts. This includes methodologies that can efficiently handle the heterogeneity inherent in MAGMA and increase the power for detecting heterogeneous effects that may provide invaluable biological insights. One of the primary methodological dilemmas in MAGMA is whether to adopt a “unified” approach, analyzing all individuals together regardless of ancestry, or a “stratified” approach, which involves separate analysis of each ancestry group, followed by MAGMA across ancestry groups. The “unified” approach may not adequately control for confounding due to population structure, while the “stratified” approach might oversimplify the complex genetic landscape by allocating individuals to rigid ancestral labels, and often overlook admixed individuals. An ideal method would synergize the benefits of both approaches by considering a continuous representation of ancestry, allowing for a more precise modeling of genetic contributions to disease across a gradient of genetic diversity.

Incorporating the cultural context of phenotype ascertainment and gene–environment interactions is currently underexplored and holds potential, especially given that traits and diseases may manifest differently across environments (Peterson et al., 2019). The development of methods accounting for these aspects is necessary to refine our understanding and treatment of disorders across diverse global populations. Additionally, exploring multiancestry interaction analysis, which allows for heterogeneity in SNP effects across ancestry groups that vary according to environment may provide important insights into the biological mechanisms that drive disease that operate differently across global populations.

## 6 CONCLUSION

GWAS and MAGMA have made significant strides in identifying genetic associations contributing to human traits, yet the journey to fully unravel the genetic underpinnings of complex diseases continues. As we venture into larger and more genetically diverse investigations, the challenges of extending observations across ancestry groups become more prominent. The future of MAGMA lies in the synergy of large‐scale, well‐designed studies, innovative analytic methodologies, and a commitment to diversity and inclusivity in genetic research.

## AUTHOR CONTRIBUTIONS

All authors contributed to writing and critical review of the manuscript.

## CONFLICT OF INTEREST STATEMENT

The authors declare no conflicts of interest.

## Supporting information

Supporting Information

## Data Availability

Data sharing is not applicable to this article as no new data were created or analyzed in this study.

## References

[ahg12572-bib-0001] Aschard, H. , Vilhjálmsson, B. J. , Joshi, A. D. , Price, A. L. , & Kraft, P. (2015). Adjusting for heritable covariates can bias effect estimates in genome‐wide association studies. American Journal of Human Genetics, 96(2), 329–339. 10.1016/j.ajhg.2014.12.021 25640676 PMC4320269

[ahg12572-bib-0002] Banda, Y. , Kvale, M. N. , Hoffmann, T. J. , Hesselson, S. E. , Ranatunga, D. , Tang, H. , Sabatti, C. , Croen, L. A. , Dispensa, B. P. , Henderson, M. , Iribarren, C. , Jorgenson, E. , Kushi, L. H. , Ludwig, D. , Olberg, D. , Quesenberry, C. P., Jr. , Rowell, S. , Sadler, M. , Sakoda, L. C. , … Risch, N. (2015). Characterizing race/ethnicity and genetic ancestry for 100,000 subjects in the Genetic Epidemiology Research on Adult Health and Aging (GERA) cohort. Genetics, 200(4), 1285–1295. 10.1534/genetics.115.178616 26092716 PMC4574246

[ahg12572-bib-0003] Begum, F. , Ghosh, D. , Tseng, G. C. , & Feingold, E. (2012). Comprehensive literature review and statistical considerations for GWAS meta‐analysis. Nucleic Acids Research, 40(9), 3777–3784. 10.1093/nar/gkr1255 22241776 PMC3351172

[ahg12572-bib-0004] Cavazos, T. B. , & Witte, J. S. (2021). Inclusion of variants discovered from diverse populations improves polygenic risk score transferability. HGG Advances, 2(1), 100017. 10.1016/j.xhgg.2020.100017 33564748 PMC7869832

[ahg12572-bib-0005] Choudhury, A. , Brandenburg, J.‐T. , Chikowore, T. , Sengupta, D. , Boua, P. R. , Crowther, N. J. , Agongo, G. , Asiki, G. , Gómez‐Olivé, F. X. , Kisiangani, I. , Maimela, E. , Masemola‐Maphutha, M. , Micklesfield, L. K. , Nonterah, E. A. , Norris, S. A. , Sorgho, H. , Tinto, H. , Tollman, S. , Graham, S. E. , … Consortium, H. A. (2022). Meta‐analysis of sub‐Saharan African studies provides insights into genetic architecture of lipid traits. Nature Communications, 13(1), 2578. 10.1038/s41467-022-30098-w PMC9095599 35546142

[ahg12572-bib-0006] DerSimonian, R. , & Laird, N. (1986). Meta‐analysis in clinical trials. Controlled Clinical Trials, 7(3), 177–188. 10.1016/0197-2456(86)90046-2 3802833

[ahg12572-bib-0007] Dias, S. , Sutton, A. J. , Welton, N. J. , & Ades, A. E. (2012). NICE decision support unit technical support documents. In Heterogeneity: Subgroups, meta‐regression, bias and bias‐adjustment. National Institute for Health and Care Excellence (NICE). Retrieved from http://www.nicedsu.org.uk 27905717

[ahg12572-bib-0008] Fatumo, S. , Sathan, D. , Samtal, C. , Isewon, I. , Tamuhla, T. , Soremekun, C. , Jafali, J. , Panji, S. , Tiffin, N. , & Fakim, Y. J. (2023). Polygenic risk scores for disease risk prediction in Africa: Current challenges and future directions. Genome Medicine, 15(1), 87. 10.1186/s13073-023-01245-9 37904243 PMC10614359

[ahg12572-bib-0009] Han, B. , & Eskin, E. (2011). Random‐effects model aimed at discovering associations in meta‐analysis of genome‐wide association studies. The American Journal of Human Genetics, 88(5), 586–598. 10.1016/j.ajhg.2011.04.014 21565292 PMC3146723

[ahg12572-bib-0010] Huang, G. H. , & Tseng, Y. C. (2014). Genotype imputation accuracy with different reference panels in admixed populations. BMC Proceedings, 8, (Suppl. 1 Genetic Analysis Workshop 18Vanessa Olmo), S64. 10.1186/1753-6561-8-s1-s64 25519397 PMC4143631

[ahg12572-bib-0011] Ishigaki, K. , Sakaue, S. , Terao, C. , Luo, Y. , Sonehara, K. , Yamaguchi, K. , Amariuta, T. , Too, C. L. , Laufer, V. A. , Scott, I. C. , Viatte, S. , Takahashi, M. , Ohmura, K. , Murasawa, A. , Hashimoto, M. , Ito, H. , Hammoudeh, M. , Emadi, S. A. , Masri, B. K. , … Raychaudhuri, S. (2022). Multi‐ancestry genome‐wide association analyses identify novel genetic mechanisms in rheumatoid arthritis. Nature Genetics, 54(11), 1640–1651. 10.1038/s41588-022-01213-w 36333501 PMC10165422

[ahg12572-bib-0012] Kamiza, A. B. , Toure, S. M. , Vujkovic, M. , Machipisa, T. , Soremekun, O. S. , Kintu, C. , Corpas, M. , Pirie, F. , Young, E. , Gill, D. , Sandhu, M. S. , Kaleebu, P. , Nyirenda, M. , Motala, A. A. , Chikowore, T. , & Fatumo, S. (2022). Transferability of genetic risk scores in African populations. Nature Medicine, 28(6), 1163–1166. 10.1038/s41591-022-01835-x PMC9205766 35654908

[ahg12572-bib-0013] Kanoni, S. , Graham, S. E. , Wang, Y. , Surakka, I. , Ramdas, S. , Zhu, X. , Clarke, S. L. , Bhatti, K. F. , Vedantam, S. , Winkler, T. W. , Locke, A. E. , Marouli, E. , Zajac, G. J. M. , Wu, K. H. , Ntalla, I. , Hui, Q. , Klarin, D. , Hilliard, A. T. , Wang, Z. , … Peloso, G. M. (2022). Implicating genes, pleiotropy, and sexual dimorphism at blood lipid loci through multi‐ancestry meta‐analysis. Genome Biology, 23(1), 268. 10.1186/s13059-022-02837-1 36575460 PMC9793579

[ahg12572-bib-0014] Kichaev, G. , & Pasaniuc, B. (2015). Leveraging functional‐annotation data in trans‐ethnic fine‐mapping studies. American Journal of Human Genetics, 97(2), 260–271. 10.1016/j.ajhg.2015.06.007 26189819 PMC4573268

[ahg12572-bib-0015] Kuchenbaecker, K. , Telkar, N. , Reiker, T. , Walters, R. G. , Lin, K. , Eriksson, A. , Gurdasani, D. , Gilly, A. , Southam, L. , Tsafantakis, E. , Karaleftheri, M. , Seeley, J. , Kamali, A. , Asiki, G. , Millwood, I. Y. , Holmes, M. , Du, H. , Guo, Y. , & Kumari, M. , … Understanding Society Scientific Group . (2019). The transferability of lipid loci across African, Asian and European cohorts. Nature Communications, 10(1), 4330. 10.1038/s41467-019-12026-7 PMC6760173 31551420

[ahg12572-bib-0016] Lam, M. , Chen, C. Y. , Li, Z. , Martin, A. R. , Bryois, J. , Ma, X. , Gaspar, H. , Ikeda, M. , Benyamin, B. , Brown, B. C. , Liu, R. , Zhou, W. , Guan, L. , Kamatani, Y. , Kim, S. W. , Kubo, M. , Kusumawardhani, A. , Liu, C. M. , Ma, H. , … Huang, H. (2019). Comparative genetic architectures of schizophrenia in East Asian and European populations. Nature Genetics, 51(12), 1670–1678. 10.1038/s41588-019-0512-x 31740837 PMC6885121

[ahg12572-bib-0017] Lewis, A. C. F. , Molina, S. J. , Appelbaum, P. S. , Dauda, B. , Di Rienzo, A. , Fuentes, A. , Fullerton, S. M. , Garrison, N. A. , Ghosh, N. , Hammonds, E. M. , Jones, D. S. , Kenny, E. E. , Kraft, P. , Lee, S. S.‐J. , Mauro, M. , Novembre, J. , Panofsky, A. , Sohail, M. , Neale, B. M. , & Allen, D. S. (2022). Getting genetic ancestry right for science and society. Science, 376(6590), 250–252. 10.1126/science.abm7530 35420968 PMC10135340

[ahg12572-bib-0018] Lin, D. Y. , & Zeng, D. (2010). Meta‐analysis of genome‐wide association studies: No efficiency gain in using individual participant data. Genetic Epidemiology, 34(1), 60–66. 10.1002/gepi.20435 19847795 PMC3878085

[ahg12572-bib-0019] Liu, J. , Lewinger, J. P. , Gilliland, F. D. , Gauderman, W. J. , & Conti, D. V. (2013). Confounding and heterogeneity in genetic association studies with admixed populations. American Journal of Epidemiology, 177(4), 351–360. 10.1093/aje/kws234 23334005 PMC3626055

[ahg12572-bib-0020] Mägi, R. , Horikoshi, M. , Sofer, T. , Mahajan, A. , Kitajima, H. , Franceschini, N. , McCarthy, M. I. , Morris, A. P. , & COGENT‐Kidney Consortium, T2D‐GENES Consortium . (2017). Trans‐ethnic meta‐regression of genome‐wide association studies accounting for ancestry increases power for discovery and improves fine‐mapping resolution. Human Molecular Genetics, 26(18), 3639–3650. 10.1093/hmg/ddx280 28911207 PMC5755684

[ahg12572-bib-0021] Mägi, R. , & Morris, A. P. (2010). GWAMA: Software for genome‐wide association meta‐analysis. BMC Bioinformatics [Electronic Resource], 11(1), 288. 10.1186/1471-2105-11-288 20509871 PMC2893603

[ahg12572-bib-0022] Mahajan, A. , Spracklen, C. N. , Zhang, W. , Ng, M. C. Y. , Petty, L. E. , Kitajima, H. , Yu, G. Z. , Rüeger, S. , Speidel, L. , Kim, Y. J. , Horikoshi, M. , Mercader, J. M. , Taliun, D. , Moon, S. , Kwak, S. H. , Robertson, N. R. , Rayner, N. W. , Loh, M. , Kim, B. J. , … Morris, A. P. (2022). Multi‐ancestry genetic study of type 2 diabetes highlights the power of diverse populations for discovery and translation. Nature Genetics, 54(5), 560–572. 10.1038/s41588-022-01058-3 35551307 PMC9179018

[ahg12572-bib-0023] Martin, A. R. , Kanai, M. , Kamatani, Y. , Okada, Y. , Neale, B. M. , & Daly, M. J. (2019). Clinical use of current polygenic risk scores may exacerbate health disparities. Nature Genetics, 51(4), 584–591. 10.1038/s41588-019-0379-x 30926966 PMC6563838

[ahg12572-bib-0024] Meng, X. , Navoly, G. , Giannakopoulou, O. , Levey, D. F. , Koller, D. , Pathak, G. A. , Koen, N. , Lin, K. , Adams, M. J. , Rentería, M. E. , Feng, Y. , Gaziano, J. M. , Stein, D. J. , Zar, H. J. , Campbell, M. L. , van Heel, D. A. , Trivedi, B. , Finer, S. , McQuillin, A. , … BioBank Japan, P. (2024). Multi‐ancestry genome‐wide association study of major depression aids locus discovery, fine mapping, gene prioritization and causal inference. Nature Genetics, 56, 222–233. 10.1038/s41588-023-01596-4 38177345 PMC10864182

[ahg12572-bib-0025] Michalek, D. A. , Tern, C. , Zhou, W. , Robertson, C. C. , Farber, E. , Campolieto, P. , Chen, W.‐M. , Onengut‐Gumuscu, S. , & Rich, S. S. (2024). A multi‐ancestry genome‐wide association study in type 1 diabetes. Human Molecular Genetics, 33, 958–968. ddae024. 10.1093/hmg/ddae024 38453145 PMC11102596

[ahg12572-bib-0026] Mills, M. C. , & Rahal, C. (2020). The GWAS diversity monitor tracks diversity by disease in real time. Nature Genetics, 52(3), 242–243. 10.1038/s41588-020-0580-y 32139905

[ahg12572-bib-0027] Morris, A. P. (2011). Transethnic meta‐analysis of genomewide association studies. Genetic Epidemiology, 35(8), 809–822. 10.1002/gepi.20630 22125221 PMC3460225

[ahg12572-bib-0028] Patrick, T. , Alicia, R. M. , Grant, G. , Hui, L. , Masahiro, K. , Raymond, K. W. , Jonathan, B. J. , Kuang, L. , Iona, Y. M. , Caitlin, E. C. , Duncan, S. P. , Meghan, Z. , Elizabeth, G. A. , Zhengming, C. , Liming, L. , Masato, A. , Yukinori, O. , Yoichiro, K. , Robin, G. W. , … Benjamin, M. N. (2021). Multi‐ancestry meta‐analysis yields novel genetic discoveries and ancestry‐specific associations, elocation‐id = 2021.04.23.441003. BioRxiv, 10.1101/2021.04.23.441003

[ahg12572-bib-0029] Peterson, R. E. , Kuchenbaecker, K. , Walters, R. K. , Chen, C. Y. , Popejoy, A. B. , Periyasamy, S. , Lam, M. , Iyegbe, C. , Strawbridge, R. J. , Brick, L. , Carey, C. E. , Martin, A. R. , Meyers, J. L. , Su, J. , Chen, J. , Edwards, A. C. , Kalungi, A. , Koen, N. , Majara, L. , … Duncan, L. E. (2019). Genome‐wide association studies in ancestrally diverse populations: Opportunities, methods, pitfalls, and recommendations. Cell, 179(3), 589–603. 10.1016/j.cell.2019.08.051 31607513 PMC6939869

[ahg12572-bib-0030] Popejoy, A. B. , & Fullerton, S. M. (2016). Genomics is failing on diversity. Nature, 538(7624), 161–164. 10.1038/538161a 27734877 PMC5089703

[ahg12572-bib-0031] Purcell, S. , Neale, B. , Todd‐Brown, K. , Thomas, L. , Ferreira, M. A. , Bender, D. , Maller, J. , Sklar, P. , de Bakker, P. I. , Daly, M. J. , & Sham, P. C. (2007). PLINK: A tool set for whole‐genome association and population‐based linkage analyses. American Journal of Human Genetics, 81(3), 559–575. 10.1086/519795 17701901 PMC1950838

[ahg12572-bib-0032] Smith, J. L. , Tcheandjieu, C. , Dikilitas, O. , Iyer, K. , Miyazawa, K. , Hilliard, A. , Lynch, J. , Rotter, J. I. , Chen, Y. I. , Sheu, W. H. , Chang, K. M. , Kanoni, S. , Tsao, P. , Ito, K. , Kosel, M. , Clarke, S. L. , Schaid, D. J. , Assimes, T. L. , & Kullo, I. J. (2024). Multi‐ancestry polygenic risk score for coronary heart disease based on an ancestrally diverse genome‐wide association study and population‐specific optimization. Circulation: Genomic and Precision Medicine, 17, e004272. 10.1161/circgen.123.004272 38380516 PMC11372723

[ahg12572-bib-0033] Sollis, E. , Mosaku, A. , Abid, A. , Buniello, A. , Cerezo, M. , Gil, L. , Groza, T. , Güneş, O. , Hall, P. , Hayhurst, J. , Ibrahim, A. , Ji, Y. , John, S. , Lewis, E. , MacArthur, J. A. L. , McMahon, A. , Osumi‐Sutherland, D. , Panoutsopoulou, K. , Pendlington, Z. , … Harris, L. W. (2023). The NHGRI‐EBI GWAS catalog: Knowledgebase and deposition resource. Nucleic Acids Research, 51(D1), D977–d985. 10.1093/nar/gkac1010 36350656 PMC9825413

[ahg12572-bib-0034] Stanley, T. D. , & Doucouliagos, H. (2015). Neither fixed nor random: Weighted least squares meta‐analysis. Statistics in Medicine, 34(13), 2116–2127. 10.1002/sim.6481 25809462

[ahg12572-bib-0035] Suzuki, K. , Hatzikotoulas, K. , Southam, L. , Taylor, H. J. , Yin, X. , Lorenz, K. M. , Mandla, R. , Huerta‐Chagoya, A. , Melloni, G. E. M. , Kanoni, S. , Rayner, N. W. , Bocher, O. , Arruda, A. L. , Sonehara, K. , Namba, S. , Lee, S. S. K. , Preuss, M. H. , Petty, L. E. , & Schroeder, P. , … Insulin‐Related Traits, C . (2024). Genetic drivers of heterogeneity in type 2 diabetes pathophysiology. Nature, 627(8003), 347–357. 10.1038/s41586-024-07019-6 38374256 PMC10937372

[ahg12572-bib-0036] Taliun, D. , Harris, D. N. , Kessler, M. D. , Carlson, J. , Szpiech, Z. A. , Torres, R. , Taliun, S. A. G. , Corvelo, A. , Gogarten, S. M. , Kang, H. M. , Pitsillides, A. N. , LeFaive, J. , Lee, S. B. , Tian, X. , Browning, B. L. , Das, S. , Emde, A. K. , Clarke, W. E. , Loesch, D. P. , … Abecasis, G. R. (2021). Sequencing of 53,831 diverse genomes from the NHLBI TOPMed program. Nature, 590(7845), 290–299. 10.1038/s41586-021-03205-y 33568819 PMC7875770

[ahg12572-bib-0037] Topriceanu, C.‐C. , Chaturvedi, N. , Mathur, R. , & Garfield, V. (2024). Validity of European‐centric cardiometabolic polygenic scores in multi‐ancestry populations. European Journal of Human Genetics, 32, 697–707. 10.1038/s41431-023-01517-3 38182743 PMC11153583

[ahg12572-bib-0038] Visscher, P. M. , Brown, M. A. , McCarthy, M. I. , & Yang, J. (2012). Five years of GWAS discovery. American Journal of Human Genetics, 90(1), 7–24. 10.1016/j.ajhg.2011.11.029 22243964 PMC3257326

[ahg12572-bib-0039] Walters, R. , Demontis, D. , Faraone, S. , Børglum, A. , & Neale, B. (2019). Increasing trans‐ethnic diversity in genome‐wide association meta‐analysis of ADHD. European Neuropsychopharmacology, 29, S722–S723. 10.1016/j.euroneuro.2017.06.039

[ahg12572-bib-0040] Willer, C. J. , Li, Y. , & Abecasis, G. R. (2010). METAL: Fast and efficient meta‐analysis of genomewide association scans. Bioinformatics, 26(17), 2190–2191. 10.1093/bioinformatics/btq340 20616382 PMC2922887

[ahg12572-bib-0041] Winkler, T. W. , Day, F. R. , Croteau‐Chonka, D. C. , Wood, A. R. , Locke, A. E. , Magi, R. , Ferreira, T. , Fall, T. , Graff, M. , Justice, A. E. , Luan, J. , Gustafsson, S. , Randall, J. C. , Vedantam, S. , Workalemahu, T. , Kilpelainen, T. O. , Scherag, A. , Esko, T. , & Kutalik, Z. , … Genetic Investigation of Anthropometric Traits (GIANT) Consortium . (2014). Quality control and conduct of genome‐wide association meta‐analyses. Nature Protocols, 9(5), 1192–1212. 10.1038/nprot.2014.071 24762786 PMC4083217

[ahg12572-bib-0042] Xiang, R. , Kelemen, M. , Xu, Y. , Harris, L. W. , Parkinson, H. , Inouye, M. , & Lambert, S. A. (2024). Recent advances in polygenic scores: Translation, equitability, methods and FAIR tools. Genome Medicine, 16(1), 33. 10.1186/s13073-024-01304-9 38373998 PMC10875792

[ahg12572-bib-0043] Yengo, L. , Vedantam, S. , Marouli, E. , Sidorenko, J. , Bartell, E. , Sakaue, S. , Graff, M. , Eliasen, A. U. , Jiang, Y. , Raghavan, S. , Miao, J. , Arias, J. D. , Graham, S. E. , Mukamel, R. E. , Spracklen, C. N. , Yin, X. , Chen, S. H. , Ferreira, T. , Highland, H. H. , … Hirschhorn, J. N. (2022). A saturated map of common genetic variants associated with human height. Nature, 610(7933), 704–712. 10.1038/s41586-022-05275-y 36224396 PMC9605867

[ahg12572-bib-0044] Zaitlen, N. , Paşaniuc, B. , Gur, T. , Ziv, E. , & Halperin, E. (2010). Leveraging genetic variability across populations for the identification of causal variants. American Journal of Human Genetics, 86(1), 23–33. 10.1016/j.ajhg.2009.11.016 20085711 PMC2801753

[ahg12572-bib-0045] Zhang, B. , Zhi, D. , Zhang, K. , Gao, G. , Limdi, N. N. , & Liu, N. (2011). Practical consideration of genotype imputation: Sample size, window size, reference choice, and untyped rate. Statistics and its Interface, 4(3), 339–352. 10.4310/sii.2011.v4.n3.a8 22308193 PMC3269888

[ahg12572-bib-0046] Zhang, R. , Kuja‐Halkola, R. , Birgegård, A. , Larsson, H. , Lichtenstein, P. , Bulik, C. M. , & Bergen, S. E. (2023). Association of family history of schizophrenia and clinical outcomes in individuals with eating disorders. Psychological Medicine, 53(2), 371–378. 10.1017/s0033291721001574 33926592 PMC9899560

