# Supplementary material for: Methods for multiancestry genome‐wide association study meta‐analysis
Source: Ann Hum Genet. 2024 Jul 18;89(5):255–63. doi: 10.1111/ahg.12572 (PMC12336937; doi:10.1111/ahg.12572)
Supplement: Supplementary file 1 — Supporting Information [file AHG-89-255-s001.docx]

APPENDIX

# Statistical Models for Standard Meta-Analysis

|  | **Weighted Z-score** | **Fixed effect** | **Random effect** | **Meta-Regression** |
| --- | --- | --- | --- | --- |
| *P*-value | √ |  |  |  |
| Effect size |  | √ | √ | √ |
| Direction of the effect size | √ |  |  |  |
| Sample size | √ |  |  | O** |
| Heterogeneity estimate |  |  | √ | O** |
| Standard Error/Variance* of effect size |  | √ | √ | √ |

Table A1: Summary statistics needed for each standard meta-analysis methods. *Variance is square of standard error. **O is for optional, as meta-regression allow for additional information for different needs

## Computing the Combined Effect Size

The two most common methods for meta-analysis are fixed-effects and random-effects, which aggregate effect sizes of multiple studies through weighted average. Weights of each study are computed differently between fixed-effects and random-effects models, which will be shown below in the respective sections.

After computing the weights of each study, the effect sizes can be aggregated using the following formula:

$$\hat{\theta_{c}}=\frac{\sum_{i=1}^{k} w_{i}\theta_{i}}{\sum_{i=1}^{k} w_{i}}$$

In this expression, $\hat{\theta_{c}}$ is the combined effect size estimates, *k* is the number of studies, $\theta_{i}$ is the effect size estimate from study *i* (aligned to the same effect allele); $w_{i}$ is the weight for study *i*.

To compute the resulting aggregate p-value, *Z-Scor*e can be computed with the following, which assumes a standard normal distribution:

$$Z=\frac{\hat{\theta_{c}}}{\text{SE}\left( \hat{\theta_{c}} \right)}$$

In this expression, $\text{SE}\left( \hat{\theta_{FE}} \right)=\sqrt{\frac{1}{\sum w_{i}}}$, is the standard error.

## Fixed-Effects Model

Fixed effect model assumes homogenous effect size across GWAS. The weights are the inverse of each study's variance which results in it to be commonly referred as inverse-variance method:

$$w_{i}=\frac{1}{\text{Var}\left( \hat{\theta_{i}} \right)}$$

In this expression, $\text{Var}\left( \hat{\theta_{i}} \right)$ is the variance of the effect size estimate from study *i*.

An alternative to this is a weighted Z-score method as used in METAL [1], also known as Stouffer’s method. This method does not require the effect size as input, as a result this is useful when effect sizes cannot be shared or are in different scales. Stouffer’s method takes p-value and direction of effect and outputs signed Z-score, using the following formula,

$$Z_{\text{weighted}}=\frac{\sum_{i=1}^{k} w_{i}Z_{i}}{\sqrt{\sum_{i=1}^{k} w_{i}}}$$

The weights, *w_i_*, are here computed as the square root of sample size of study *i*; Z-score here is computed from the p-values and direction of effect of the study as such,

$$Z_{i}=\Phi^{-1}\left( 1-\frac{P_{i}}{2} \right)\times\left( \text{direction of effect for study }i \right)$$

In this expression, $P_{i}$ is p-value of study *i*, and $\Phi^{-1}$ is inverse of the cumulative distribution function.

# Heterogeneity

Two common ways for assessing heterogeneity between studies are Cochran's Q statistic and I² statistic. Their formula is as follow, Cochran's Q statistic is computed as such,

$$Q=\sum_{i=1}^{k} w_{i}\left( \hat{\theta_{i}}-\hat{\theta_{FE}} \right)^{2}$$

In this expression, $\theta_{FE}$ is the combined effect size estimate under the fixed effects model.

As an alternative, the *I*² statistic, describes the percentage of variation across studies not attributable to chance, the following is the formula:

$$I^{2}=\max\left( 0,\frac{Q-\left( k-1 \right)}{Q}\times100\% \right)$$

## Random-Effects Model

The random-effects model, in contrast, recognizes the existence of variability in the true effects across various studies, implying that effect sizes are follow a distribution across studies. This approach allows for testing of the null hypothesis that the true effect size across GWAS is zero, which would also help identify if an effect is study or in the case of MAGMA, ancestry specific. This model is particularly pertinent in multi-ancestry GWAS, where genetic diversity is inherent. It accounts for both within-study variance and between-study variance, thus providing a more nuanced estimation when heterogeneity is substantial. To achieve this random-effects approach uses the following weights by DerSimonian and Laird [2].

$$w_{i}^{*}=\frac{1}{\text{Var}\left( \hat{\theta_{i}} \right)+\tau^{2}}$$

In this expression, $\tau^{2}$ is the between between-study variance and is as computed as such,

$$\tau^{2}=\max\left( 0,\frac{Q-\left( k-1 \right)}{\sum w_{i}-\frac{\sum w_{i}^{2}}{\sum w_{i}}} \right)$$

where *k* is the number of studies, *w_i_* is the within-study weight as computed in the fixed-effects model and *Q* is Cochran's Q statistic as defined above. This approach not only gives a pooled estimate of the effect size but also allows for the exploration of the distribution of these effects across studies, making it invaluable for MAGMA.

## Meta-Regression

Meta-regression extends beyond the above two methods using linear regression framework. where additional variables can be incorporated to help identify how different variables might influence the effect sizes observed, i.e. contribute to heterogeneity. In typical meta-analysis it would be demographic characteristics, study designs, or environmental factors. In the case of multi-ancestry, genetic ancestry information could be incorporated into the model just as was done in MR-MEGA, which calculates the genetic variation using GWAS being meta-analysed [3]. A simple meta-regression would be as follows,

$$Y_{i}=\beta_{0}+\beta_{1}X_{i1}+\beta_{2}X_{i2}+\cdots+\beta_{k}X_{ik}+\epsilon_{i}$$

In this expression, $Y_{i}$*,* represents the dependent variable (effect sizes) in the meta-regression for study *i* aligned to the same effect allele*;* $\beta_{0}, \beta_{1}$*,* … $\beta_{k}$ are the intercept and slope coefficients in the meta-regression; $X_{i1}, X_{i2},$ … $X_{ik}$ would be variables to account for that may contribute to heterogeneity; $\epsilon$is the error term in the meta-regression which would be weighted.

Just as linear regression has variations from fixed effect regression to mixed effects regression, these are applicable to meta-regression as well. One variation of the regression framework has been suggested to outperform both fixed and random effects method when number of studies being analysed is small, which is the weighted least square regression model. This model allows for variation per study to be accounted for [4, 5], where the weight is inverse variance of each study’s effect size.

Those familiar with Bayesian statistics would be right to assume random-effects and meta-regression could be easily extended to apply probabilistic modelling and expanded with hierarchical versions of it. While not covered in this review, interested readers can refer to tutorial by Reis et al. for an in-exploration of random-effects Bayesian meta-analyses [6].

# REFERENCES

1. Willer CJ, Li Y, Abecasis GR: **METAL: fast and efficient meta-analysis of genomewide association scans**. *Bioinformatics* 2010, **26**(17):2190-2191.

2. DerSimonian R, Laird N: **Meta-analysis in clinical trials**. *Control Clin Trials* 1986, **7**(3):177-188.

3. Mägi R, Horikoshi M, Sofer T, Mahajan A, Kitajima H, Franceschini N, McCarthy MI, COGENT-Kidney Consortium TD-GC, Morris AP: **Trans-ethnic meta-regression of genome-wide association studies accounting for ancestry increases power for discovery and improves fine-mapping resolution**. *Human Molecular Genetics* 2017, **26**(18):3639-3650.

4. Stanley TD, Doucouliagos H: **Neither fixed nor random: weighted least squares meta-analysis**. *Statistics in Medicine* 2015, **34**(13):2116-2127.

5. Dias S, Sutton AJ, Welton NJ, Ades AE: **NICE Decision Support Unit Technical Support Documents**. In: *Heterogeneity: Subgroups, Meta-Regression, Bias And Bias-Adjustment.* London: National Institute for Health and Care Excellence (NICE)

Copyright © 2012 National Institute for Health and Clinical Excellence, unless otherwise stated. All rights reserved.; 2012.

6. Reis DJ, Kaizer AM, Kinney AR, Bahraini NH, Holliday R, Forster JE, Brenner LA: **A practical guide to random-effects Bayesian meta-analyses with application to the psychological trauma and suicide literature**. *Psychol Trauma* 2023, **15**(1):121-130.
